# Identification of Newer Stable Genetic Sources for High Grain Number per Panicle and Understanding the Gene Action for Important Panicle Traits in Rice

**DOI:** 10.3390/plants12020250

**Published:** 2023-01-05

**Authors:** Ariharasutharsan Gunasekaran, Geetha Seshadri, Saraswathi Ramasamy, Raveendran Muthurajan, Krishna Surendar Karuppasamy

**Affiliations:** 1 Department of Rice, Centre for Plant Breeding and Genetics, Tamil Nadu Agricultural University, Coimbatore 641003, India; 2 Department of Pulses, Centre for Plant Breeding and Genetics, Tamil Nadu Agricultural University, Coimbatore 641003, India; 3 Department of Plant Genetic Resources, Centre for Plant Breeding and Genetics, Tamil Nadu Agricultural University, Coimbatore 641003, India; 4 Department of Plant Biotechnology, Centre for Plant Molecular Biology and Biotechnology, Tamil Nadu Agricultural University, Coimbatore 641003, India; 5 Department of Seed Science and Technology, Agricultural College and Research Institute, Tamil Nadu Agricultural University, Madurai 625104, India

**Keywords:** rice, combining ability, D^2^ analysis, grain number per panicle, PCA

## Abstract

Rice is an important food crop extensively cultivated worldwide, and rice’s grain yield should be improved to meet future food demand. Grain number per panicle is the main trait that determines the grain yield in rice, and other panicle-related traits influence the grain number. To study the genetic diversity, 50 diverse Indian-origin germplasm were evaluated for grain number per panicle and other panicle traits for two consecutive seasons (*Rabi* 2019 and *Kharif* 2020). The maximum genotypic and phenotypic coefficient of variation was obtained for the number of spikelets and filled grains per panicle. The genotypes were grouped into eight clusters with Mahalanobis’ D^2^ analysis and six groups using Principal component analysis. Based on, per se, performance for grain number per panicle and genetic distances, six parents were selected and subjected to full diallel mating. The genotypes CB12132, IET 28749, and BPT 5204 were the best general combiners for the number of filled grains per panicle and other panicle branching traits, viz., the number of primary and secondary branches per panicle. The hybrid BPT 5204 × CB 12132 identified as the best specific combination for most of the studied panicle traits. The additive gene effects were high for the number of filled grains per panicle, the number of primary branches, and secondary branches, whereas non-additive gene action was high for the number of productive tillers and grain yield per plant. The information obtained from this study will be useful in rice breeding programs to improve panicle traits, especially the grain number, which would result in higher grain yield.

## 1. Introduction

Rice (*Oryza sativa* L.) is an important food crop that is extensively grown around the world. It contains a rich source of energy, 80% of carbohydrates and starch, 7% of proteins with a high amount of glutamic and aspartic acid, and vitamins E (tocopherol), B_1_ (thiamine), and B_3_ (niacin) [1]. Rice is cultivated in over 100 countries, covering around 158 million hectares of area and producing more than 700 million tons annually [2]. However, Asia alone accounts for 90% of the world’s rice production, whereas the remaining 10% is produced by Sub-Saharan Africa and Latin America [3]. India is one of the largest rice producers in the world and accounts for around one-third of global rice production. Rice is mainly cultivated in the Indian states, including Andhra Pradesh, Tamil Nadu, Karnataka, West Bengal, Uttar Pradesh, Orissa, and Chhattisgarh [4]. There is a dire need to increase the rice yield proportionally to feed the ever-increasing population, which rises in geometrical proportion. Hence, improving grain yield remains the foremost objective in the rice breeding programs in India. The grain yield of rice is determined by the number of panicles per plant, grain number per panicle, and grain weight [5]. Of these, grain number per panicle (GNPP) is an important trait in determining grain yield and a major trait of concern for developing new plant types in rice [6,7].

India is the bedrock of rice diversity, with unique collections of germplasms that could be a valuable genetic resource for future crop improvement programs to meet the increasing demand for food production. Knowledge of the genetic diversity of the germplasm is the prerequisite to choosing the parents for recombination breeding aimed at improving any trait. Crossing among the accessions belonging to diverse clusters/groups showed desirable recombinants in segregating generations [8]. Mahalanobis’ D^2^ and principal component analysis (PCA) are the powerful pre-breeding tools used to select the diverse germplasm for hybridization and to estimate each germplasm’s potentialities [9]. The combined use of these two techniques was attempted recently in rice accessions to assess the phenotypic diversity of various traits by Dhakal, Pokhrel, Sharma, and Poudel [8]; Tiwari et al. [10]; and Lakshmi et al. [11] For genetic analysis in breeding, different mating designs are being followed, viz., Line × Tester (L × T), biparental cross, polycross, North Carolina design (I, II and III), and diallel (I, II, III, and IV) [12,13,14,15]. Each design has its own advantages, but Griffing’s full diallel mating design uses a general linear model, which is a widely used technique to identify the genetic and maternal effect of specific traits and for choosing parents and hybrids with general and specific combining ability, respectively, in the desired direction, which can be deployed in plant breeding [16].

Therefore, the present study was undertaken with the following objectives: (1) to assess the phenotypic diversity of rice germplasm and selection of parents based on, per se, performance and genetic distance and (2) genetic and combining ability analyses of parents for panicle- and yield-related traits. 

## 2. Results

### 2.1. Per Se Performance and Genetic Variability

The diverse rice genotypes exhibited notable variation for all the panicle- and yield-related traits. The pooled analysis of variance (ANOVA) found that the genotypes mean sum of square was significant (*p* ≤ 0.05) for all the studied traits, and genotypes × season interaction was also significant for all the traits except the number of secondary branches per primary branch (Table S1). Mean, range, variability, heritability, and genetic advance as percentages of the mean for the number of filled grains and other panicle- and yield-related traits were given in Table 1.

The number of spikelets per panicle and the number of filled grains per panicle ranged from 40 to 430 and 30.5 to 409.5, with averages of 228.59 ± 6.725 and 211.29 ± 7.535, respectively. The number of filled grains per panicle was recorded as more than 300 for eight genotypes, viz., CB12132, IET 28749, IET 29529, IET 29537, KNM 7714, NWGR–16022, IET 29539, and KNM 7715, while it was lower than 100 for two genotypes, ACM -20001 and IET 28835. The genotype CB12132 registered the maximum mean for the number of spikelets and filled grains per panicle (430 and 409.5), and mutant ACM-20001 recorded the lowest number of spikelets and filled grains per panicle (40 and 30.5). 

**Figure 1:**
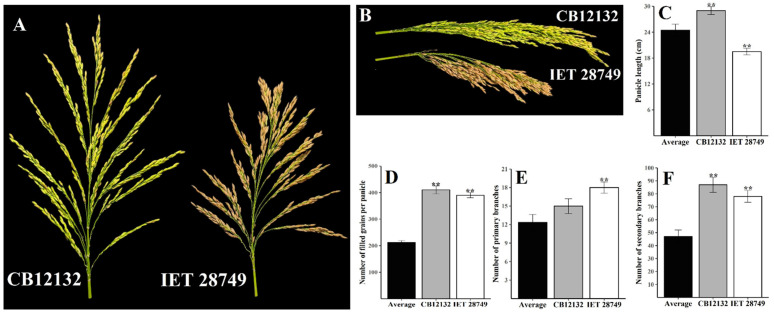
Two new genotypes, i.e., CB12132 and IET 28749, identified for the high number of filled grains per panicle and superior panicle characteristics. (**A**–**B**) panicle morphology of CB12132 and IET 28749. (**C**–**F**) Comparison of panicle length, number of spikelets per panicle, number of filled grains per panicle, number of primary branches, and number of secondary branches in CB12132 and IET 28749 with the average value of 50 germplasm over two seasons. Error bar shows standard error (*n* = 10); ** indicate 1% significant difference from average compared to the Student’s *t*-test.

Concerning the panicle branching characters, the number of primary branches ranged from 7.5 to 18.5 with an average of 12.35 ± 0.59, and the number of secondary branches ranged from 10.5 to 87 with an observed average of 47.06 ± 2.37. The genotypes CB12132, IET 29537, and IET 28749 produced the maximum number of secondary branches (87, 81.5, and 78), and a higher number of primary branches was found in IET 29537 (18.5), *Kothamalli samba* (17.5), and IET 28749 (17) (Figure 1). The number of spikelets arranged in primary branches ranged from 22 to 113, and those in secondary branches ranged from 18 to 357 with averages of 62.14 and 166.45. 

Regarding grain yield per plant, CO (R) 50 and *Kothamalli samba* exhibited the highest values, 52.5 g and 48.935 g, respectively; meanwhile, low values were observed in ACM 20001 (3.5 g) and IET 28834 (11.75 g). For plant height, it varied from 30 cm to 163.5 cm with an average of 100.07 ± 2.47, where landraces, viz., *Sembil priyan* (163.5 cm), *Kothamalli samba* (154 cm), *Karuka* (141 cm), *Mikkuruvai* (134.5 cm), and *Katta samba* (127.5 cm) occupied the top five ranks. On the other hand, the traits number of productive tillers, flag leaf area, and panicle length values ranged from 8.5 to 42.5 nos. (IET 29529 to *Muttakar*), 3.5 to 62.19 cm^2^ (ACM- 20001 to *Kallukar*), and 8.5 to 29.25 cm (ACM 20001 to CO (R) 50), respectively.

The level of GCV, PCV, heritability (h^2^), and GASM for fourteen traits are presented in Table 1. The GCV values ranged between 13.96 to 45.71%, where high GCV (>20%) was recorded for 11 traits, and moderate GCV was found in panicle length (13.96%), flag leaf area (14.19%), and length of primary branches (15.36%). As for PCV, it ranged from 14.63% in panicle length to 47.47% in the number of spikelets in secondary branches. The broad sense heritability value recorded a maximum (>60%) for all the traits. GASM values ranged from 27.44% in panicle length to 90.67% in the number of spikelets in secondary branches. High PCV, GCV, h^2^, and GASM coupled in eleven traits, showing the role of high additive gene effects and high genetic gain, which would allow selection in the early segregating generation to achieve a maximum genetic gain in recombination breeding.

### 2.2. Genetic Diversity and Principal Component Analysis 

According to Mahalanobis’ D^2^ statistics, the phenotypic distance matrix was constructed among 50 genotypes using the 14 morphometric observations, which included the number of filled grains per panicle and other panicle traits. In this case, using the toucher clustering technique, genotypes were grouped into six clusters with a cut-off value of 1543.457. The intra and inter-cluster distances of clusters derived from computed D^2^ analysis showed the statistical difference among 50 rice genotypes. Cluster I was considered the largest cluster, comprising 29 genotypes, followed by cluster II, containing 11 genotypes. Concerning other clusters, seven genotypes were in cluster III and only one genotype in the solitary clusters IV (IET 28749), V (IET 29537), and VI (ACM-20001) (Table 2). Most of the landraces were grouped in cluster I and cluster II along with some released varieties and genetically stabilized cultures, which might be due to the close genetic relationship among themselves. Further, the grouped landraces might have the same region of origin or different ecotypes.

The maximum cluster means for various traits were recorded as follows: plant height (106.052 cm) in cluster I, length of primary branches (11.764 cm) and thousand-grain weight (21.946 g) in cluster II, flag leaf area (42.15 cm^2^), number of spikelets per panicle (417.5 nos.), number of filled grains per panicle (389.5 nos.) and number of spikelets in secondary branches (348.5 nos.) in cluster IV, panicle length (25.9 cm), number of primary branches (18.5 nos.), number of secondary branches (106.5 nos.), number of secondary branches per primary branch (5.765 nos.), number of spikelets in primary branches (102 nos.), grain yield per plant (34.83 g) in cluster V, and number of productive tillers (35 nos.) in cluster VI (Table 3).

The maximum intra-cluster distance was found in cluster III (1244.3348), followed by cluster II (750.9652), cluster I (635.2865), and the rest of the clusters were solitary with no intra-cluster distance. In the case of inter-cluster distances, the maximum inter-cluster distance was recorded between cluster II and cluster IV (17,413.5), indicating the distant relation among themselves, followed by cluster II and V (12,616.88), cluster I and IV (11,524.82), cluster IV and VI (8143.019), and cluster I and V (7891.748) (Table 4). Hence, genotypes of cluster II with cluster IV and V can be crossed to exploit transgressive segregants for the panicle traits due to their diverse nature. 

A total of 50 genotypes raised in *Rabi* 2019 and *Summer* 2020 were used for PCA analysis. For both seasons, consistent PCA results were obtained. For results in *Summer* 2020, PC1, PC2, and PC3 components covered 72.104% of the total variation and recorded an eigenvalue of more than one. Other PC components with an eigenvalue of less than one were ignored because they are unlikely to have any practical significance. The PC1 component includes 47.333% of total variation and had positive loading for all the studied traits (0.145 in LOPB to 0.969 in NOSISB) except for plant height (−0.01), the number of productive tillers (−0.135), and thousand-grain weight (−0.305). These results indicated that genotypes with high PC1 scores have more spikelets and filled grains per panicle with desirable panicle characteristics. The PC2 component recorded a negative loading value for the number of productive tillers (−0.03), the number of secondary branches (−0.036), and number of secondary branches per primary branch (−0.074), and the rest of the traits had positive loadings (0.016 in TGW to 0.923 in PH) (Table 5). 

**Figure 2:**
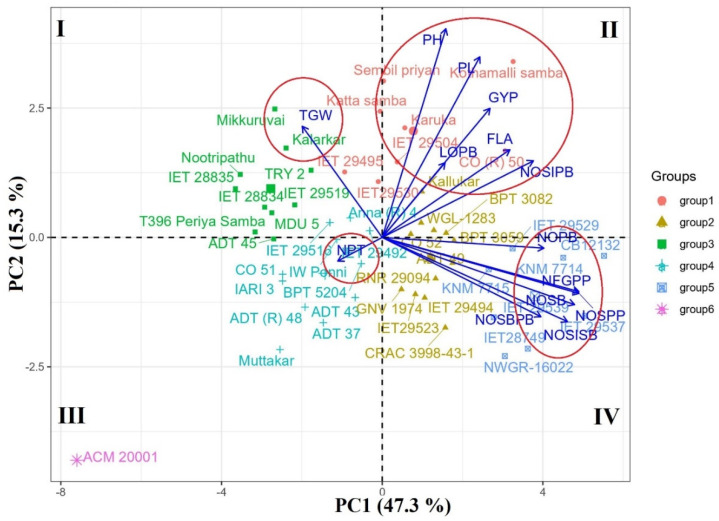
PCA biplot of 50 rice accessions and 14 traits plotted by PC1 versus PC2 component.

From the PCA biplot, the genotypes with positive PC1 and PC2 loading values were placed on quadrant 2, and genotypes with positive PC1 and negative PC2 loading were located on quadrant 4 (Figure 2). Thus, genotypes in quadrant 2 had positively higher values for panicle length, number of spikelets in primary branches, and grain yield per plant, i.e., CO (R) 50 and *Kothamalli samba*; and genotypes in quadrant 4 exhibited higher values for the number of spikelets, filled grains, primary branches, and secondary branches per panicle, i.e., CB12132, IET 28749, IET 29537, NWGR-16022, etc. The genotypes classified under groups 1, 2, and 5 had superior panicle characteristics and grain yield per plant. Genotypes with higher thousand-grain weight were placed in quadrant 1, i.e., *Mikkuruvai, Kalarkar*, TRY 2, *Nootripathu*, etc., and genotypes with an increased number of productive tillers were placed in quadrant 3, viz., Co 51, IW Ponni, BPT 5204, etc. These genotypes may be used as parents in rice breeding along with genotypes with desirable panicle characters that exhibit segregants with enhanced grain yield. The mutant ACM 20001 was highly divergent for all the studied characters with other genotypes due to its dwarfing nature. In the case of trait association, the four encirclings made in the biplot showed that grain yield per plant had a close association with plant height, panicle length, length of primary branches, flag leaf area, and the number of spikelets in primary branches. The number of filled grains per panicle had a close vector angle with the number of spikelets per panicle and the number of secondary branches. However, the thousand-grain weight and number of productive tillers had a negative association with most of the panicle traits.

### 2.3. Correlation among Panicle- and Yield-Related Traits

**Figure 3:**
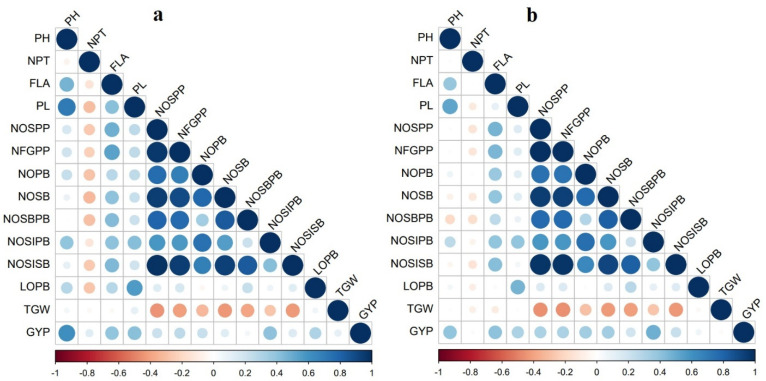
Pearson’s correlation matrix for the panicle- and yield-related traits studied during (**a**) *rabi* 2019 and (**b**) *summer* 2020.

A positive correlation was found between the number of filled grains per panicle and grain yield per plant (r = 0.26 and 0.32, *p* < 0.01). The panicle traits, viz., number of spikelets per panicle, number of primary branches, number of secondary branches, number of spikelets in primary branches, number of spikelets in secondary branches, and length of primary branch, had a significant positive association with grain yield per plant and number of filled grains per panicle. It showed that improving panicle characteristics is essential for enhancing the grain yield in rice. Flag leaf area had a significant, highly positive correlation with number of filled grains per panicle (r = 0.54 and 0.46, *p* < 0.01) and grain yield per plant (r = 0.40 and 0.45, *p* < 0.01). The number of productive tillers and thousand-grain weight recorded a significant negative correlation with all the panicle characters in both seasons (Figure 3).

### 2.4. Diallel Analysis

Based on the (1) genetic distance obtained from D^2^ and PCA analysis and (2) per se performance of germplasm, the following six genotypes were selected for low (L), medium (M), and high (H) categories of the number of filled grains per panicle: IET 28834 (L), IET 28835 (L), ADT (R) 48 (M), BPT 5204 (M), CB12132 (H), and IET 28749 (H) and thirty hybrids were generated using full diallel mating design. The results of the analysis of variance for 6 genotypes and their 30 F_1_ hybrids tested via diallel fashion (including reciprocals) are depicted in Table 6. 

The mean sum of squares was found significant for all traits studied, indicating the existence of potential genetic variance in the genotypes employed in this investigation. The analysis of variance for combining ability suggested that the mean sum of the square for GCA and SCA was commonly significant for all the traits except SCA, which was not significant for the number of secondary branches per primary branch. The significant difference in RCA stipulates the reciprocal effects of cross-combinations in traits, viz., plant height, panicle length, number of secondary branches per primary branch, and grain yield per plant (Table 6).

The determination of elite parents and desirable cross-combinations lies greatly in the magnitude and direction of combining ability effects. The estimates of general ability effects for studied traits are given in Table 7. For the most prime trait, grain yield per plant, parents, viz., IET 28749 (4.488) and BPT 5204 (1.266), showed a significant positive GCA effect.

The genotypes CB12132, IET 28749, and BPT 5204 exhibited highly significant positive GCA effects for panicle-related traits like the number of spikelets per panicle, number of filled grains per panicle, number of primary branches per panicle, number of secondary branches per panicle, number of secondary branches per primary branch, number of spikelets in primary branches, number of spikelets in secondary branches, along with grain yield per plant. In the case of the length of the primary branch, CB12132 recorded the highest GCA effect, followed by IET 28749. Among the parents, CB12132 exhibited a superior GCA effect for the number of filled grains per panicle (88.514) and also had a high mean value (423 ± 15 nos.). It showed that CB12132 would be a good general combiner for the number of filled grains per panicle, and other panicle traits could be utilized as a donor in rice breeding. In the case of other major traits, IET 28749 for grain yield per plant, ADT (R) 48 for the number of productive tillers, and IET 28835 for thousand-grain weight had higher GCA effects. The segregants for high grain number along with more tillering and high grain weight can be identified using these genotypes as a donor in rice breeding.

The dominance and interaction effects between the parental genotypes result in the high specific combining ability. The estimates of specific combining ability for 15 hybrids and their reciprocals are depicted in Table S2. Among the set of diallel hybrids, IET 28834 × BPT 5204, CB12132 × IET 28834, CB12132 × IET 28835, and ADT (R) 48 × CB 12132 exhibited significant positive SCA effects for grain yield per plant. Considering the panicle-related traits, the hybrid BPT 5204 × CB12132 was a good specific combination for panicle length, number of spikelets per panicle, number of filled grains per panicle, number of secondary branches, number of spikelets in secondary branches, and length of the primary branch, with high mean value considered as the elite specific combiners. IET 28749 × CB12132 exhibited the highest specific combining ability for the number of spikelets in primary branches (10.625) with the high mean value for the number of filled grains per panicle. IET 28834 × IET 28835 recorded a high positive SCA effect for most panicle-related traits but exhibited a lower mean value. 

**Figure 4:**
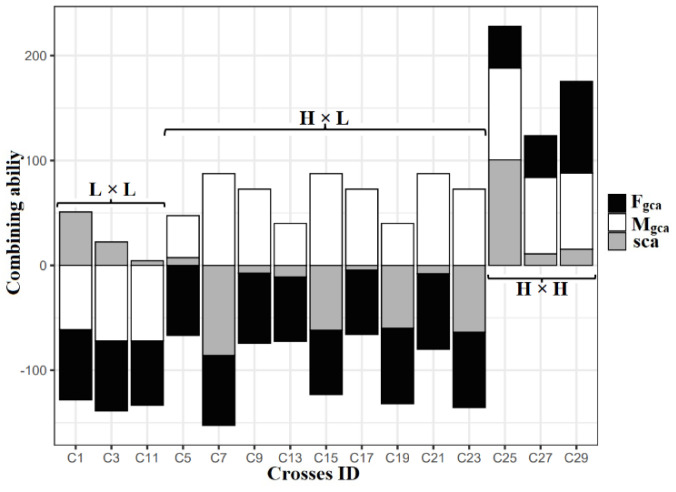
Comparison of the relationship between parental GCA and F_1_’s SCA on the number of filled grains per panicle for 15 direct crosses. F_gca_, GCA effect of the female parent; M_gca_, GCA effect of the male parent; SCA, SCA effect of F_1_s; L, low GCA; H, high GCA.

The SCA of direct crosses (15 hybrids) compared with the parental GCA for the number of filled grains per panicle and the results showed that three crosses had low × low GCA, nine crosses had high × low GCA, and three crosses had high × high GCA combinations. Interestingly, the crosses derived from the low × low GCA exhibited positive SCA on its hybrids among IET 28834 × IET 28835, which was significant. In the case of high × low GCA combinations, none of the crosses showed significant positive SCA. For high × high combination, no negative SCA was observed, and a cross BPT 5204 × CB12132 recorded significant positive SCA for the number of filled grains per panicle (Figure 4).

**Figure 5:**
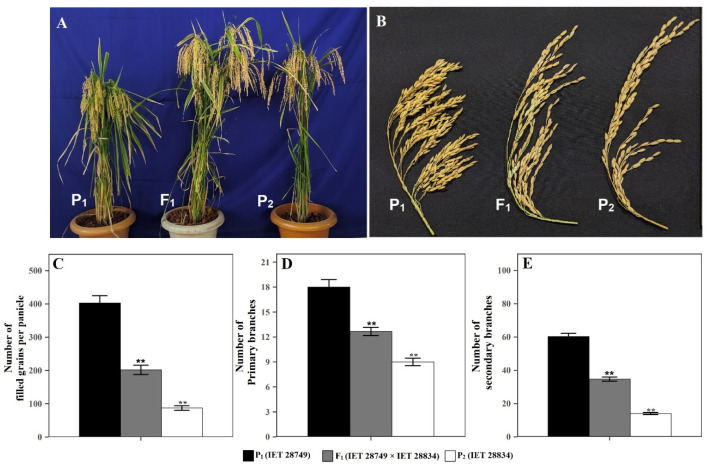
Phenotypic comparison of (**A**) plant, (**B**) panicle characteristic, (**C**) number of filled grains per panicle, (**D**) number of primary branches, and (**E**) number of secondary branches in High × Low cross (IET 28749 × IET 28834) (P_1_ = IET 28749, P_2_= IET 28834, F_1_ = IET 28749 × IET 28834). Error bar shows standard error (*n* = 10); ** indicate significant difference from parent 1 (IET 28749) as compared by the Student’s *t*-test.

Phenotypic comparison of IET 28749 × IET 28834 (high × low mean) hybrid and its parents showed that F_1_ had the intermediate mean value between the two parents for the number of filled grains per panicle, number of primary branches, and number of secondary branches (Figure 5). 

**Figure 6:**
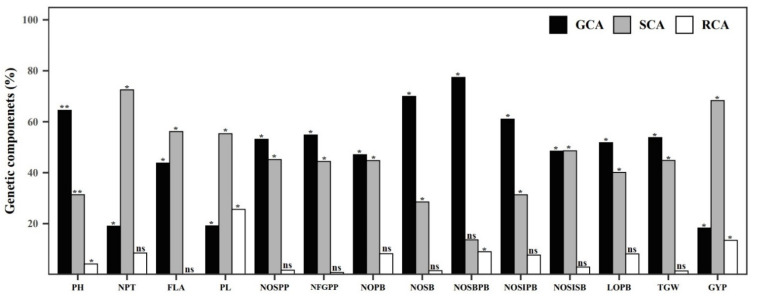
Percentage of genetic components present in fourteen panicle- and yield-related traits estimated by Griffing’s diallel analysis method I model I. * and ** indicates 5% and 1% significant difference from average compared to the Student’s *t*-test, ns indicates not significant.

GCA and SCA variance exhibited the influence of additive and dominant gene action. The GCA/SCA variance for the number of productive tillers, panicle length, and grain yield per plant was recorded as lower than one and showed the presence of high non-additive gene action in those traits. Most of the panicle-related traits and plant height had a GCA/SCA variance greater than one, indicating the presence of high additive gene action (Table 6). The higher proportion of RCA variance was found in panicle length (25.584%), length of secondary branches (16.360%), and grain yield per plant (13.438%). The higher GCA variance was present in the number of secondary branches per primary branch (77.392%). A higher proportion of SCA variance was present in the number of productive tillers (87.316%), grain yield per plant (63.392%), and flag leaf area (55.84%) (Figure 6).

## 3. Discussion

The present study explored the genetic variability of 50 rice germplasms originating from India. In experiment 1, we evaluated 14 characters in 50 rice germplasms for two consecutive seasons, viz., *Rabi* 2019 and *Summer* 2020, and the combined analysis for variance and genetic parameters over two seasons was calculated [17,18]. 

The genotypes CB12132, IET 28749, NWGR-16022, IET 29529, IET 29539, KNM 7714, and KNM 7715 exhibited more than 300 filled grains per panicle, with a wide range of panicle lengths of 18.5 cm to 28.9 cm, thus exhibiting the wide variability in panicle architecture of the observed high-NFGPP genotypes. The high NFGPP in rice with the following donors was previously reported: 187.8 ± 12 (Teqing), 220 ± 10 (9311), 230 ± 15 (Guichao 2), and 280 ± 20 (FAZ1) [19,20,21]. The number of primary and secondary rachis branches is positively associated with grain number per panicle and grain yield. In the case of our genotypes, four had more than 17 NOPB, and seven genotypes had more than 70 NOSB, which was high compared to the previous reports: 12.5 ± 0.8, 13.7 ± 1.1 and 15 ± 1.4 for NOPB and 35 ± 3.6, 54.9 ± 4.7, and 69 ± 3.2 for NOSB [22,23,24]. The higher panicle branching in rice is relatable to the ability of genotypes to form more axillary meristem from the main rachis meristem and primary rachis meristem. It is supported by the activity of various hormones, viz., disruption in auxin synthesis and transport reduces NOSB and NOSPP [25], the gain of function of *OsCYP71D8L* in inflorescence meristem related to gibberellins reduces NOSPP and PL [26], and the well-known *OsCKX2* gene degrades cytokinin and negatively regulates NOSPP [27]. The present study highlights the importance of the high NFGPP in improving grain yield, and it is supported by previous studies that proved that improvement of grain yield in rice is possible via introgression of grain number QTLs, viz., *Gn1a*, *OsSPL14*, and *qGN 4.1*, to low-yielding genotypes [28,29,30,31,32] The high- and low-yielding genotypes were differentiated based on the number of filled grains per panicle and the number of rachis branches as the primary determinant, thereby showing that the importance of the number of filled grains per panicle (>300) could be treated as one of the major selection criteria for improvement of yield [33,34,35]. In the case of other major traits, CO (R) 50 and *Kothamalli samba* for GYP, KNM 7714 for NPT, and IET 29529 for TGW had higher values without compromising the superior panicle characteristics and yield.

Among the traits studied, the NOSPP and the NFGPP exhibited maximum phenotypic and genotypic coefficient of variation, thus ensuring their significance in utilization for grain yield improvement breeding programs [36,37]. The magnitude of the difference between GCV and PCV shows the amount of environmental influence on these traits, and the low difference refers to the occurrence of strong genetic effects [38,39]. The results showed that high GCV was obtained for 11 traits except for LOPB, FLA, and PL due to increased genetic variation, and improving these traits could be more effective. Similarly, previous reports also showed high-to-moderate GCV and PCV for NPT, NFGPP, TGW, and GYP [36,40]. Genetic advance coupled with high heritability is a more reliable measure for determining the usefulness of selection on the traits. Related to our study, high h^2^ and GASM combination was observed for the major traits, viz., PH, NFGPP, NOPB, NOSB, TGW, and GYP [37,41,42], and thereby allowed for selection in segregating generations in conventional recombination breeding methods.

In Mahalanobis’ D^2^ analysis, six clusters were formed, and cluster I comprised a maximum of 29 genotypes, displaying its relatedness. Clusters IV, V, and VI had single genotypes that showed their uniqueness for studied traits. The maximum genetic distance was observed between cluster II and clusters IV, where 11 genotypes in cluster II had the common features of low panicle traits and were mixed with popular varieties, landraces, and pre-released cultures; and cluster IV had IET 28749 found for desirable panicle characters, and these highly divergent genotypes can be used as parents in hybridization and selection programs. The distant relationship between landraces and varieties by the pattern of grouping, where most of the landraces were grouped in clusters I and II and where cluster I ranked first for average plant height, explained the tall nature of landraces. At the same time, this is relatable to previous studies [11,43]. CB12132, CRAC 3998-43-1, NWGR-16022, IET 29529, IET 29539, KNM 7714, and KNM 7715 in cluster III; IET 28749 in cluster IV; and IET 29537 in cluster V had a high mean value for NFGPP, and GYP suggests that employing these genotypes in plant breeding programs greatly enhances grain number per panicle and improves grain yield [44].

Principal component analysis (PCA) reduces the dimensionality of the data set by creating significant principal components, contributing to the maximum variability of the genotypes. In PCA, standardization of data made attributes contribute equally towards the divergence studies irrespective of the units taken [45]. In our study, three principal components compiled 72.104% of the total variation for fourteen characters. The genotypes scattered into different quadrants (I, II, III, and IV) showed the high genetic variation for NFGPP and other panicle- and yield-related traits present in the genotypes. PC1 and PC2 components had positive loading values for most panicle-related traits. Both have negative loading for NPT, showing the opposite relationship between panicle characters and NPT, but TGW had negative loading in PC1 and positive in PC2 [37]. 

From the PCA biplot, the group 2 and group 5 genotypes exhibit low tillering, low TGW, and high panicle trait value and grain yield per plant, and genotypes in group 1 exhibit desirable panicle characters along with high TGW and high grain yield per plant. The selection of low tillering and higher panicle characters leads to a plant type similar to IRRI’s new plant type verities, which had thick and strong culm, low tillers and 100% productive tillers, large panicles, vigorous root system, and high yield [46]. The high tillering in rice is related to increased biomass and exhibited dense canopy, which facilitates the microenvironment and is amenable to pests and diseases. Late-formed tillers had small panicles and poor grain filling also, and low tillers had insufficient panicles, which decreased grain yield. Therefore, obtaining low tillers, broad panicles with high grain numbers, and gain filling will be suitable for desirable plant types in rice [47]. The genotypes, i.e., ACM 20001, *Kothamalli samba, Mikkuruvai*, and NWGR-16022, which were plotted more away from origin, had highly diverse from other genotypes, which can be used in hybridization to ensure wide genetic variability in the recombinants. Nahar et al. [48] revealed that four principal components together accounted for 77.94% of the variation among 50 rice genotypes. The results of both multivariate techniques showed a more or less similar pattern of grouping genotypes. Clusters III, IV, and V of Mahalanobis’ D^2^ analysis and group 1, 2, and 5 genotypes of the PCA biplot significantly impacted panicle characters and grain yield per plant [11,49]. From PCA, Co (R) 50 and *Kothamalli samba* were selectively identified for their high-yielding nature and desirable panicle traits, which were also grouped in cluster I of D^2^ analysis.

From Pearson’s correlation coefficient analysis of two seasons, it was understood that all the panicle characteristics, including the number of filled grains per panicle, need to be improved to enhance the grain yield due to its strong positive association. The flag leaf area is positively associated with both panicle characters and grain yield, so it should be enhanced to improve both NFGPP and GYP simultaneously. The high leaf area was relatable with a high photosynthetic rate and transpiration rate, expanding the grain filling period [50]. The negative association of NPT and TGW with GYP showed that the negative relationship among them and selection towards high GNPP lead to a plant type with low tillering and high grain filling. These plant types could be similar to the IRRI’s new plant type and have been reported as desirable in previous studies [51]. Tu Anh et al. [52] also reported the negative association of TGW with panicle characters, similar to our research.

### Diallel Analysis

Improvement of panicle-related traits is important to enhance the grain yield in rice. We identified that GCA and SCA variances were significant (*p* ≤ 0.05) for all the traits except the number of secondary branches per primary branch. This suggests the role of both additive and non-additive effects on the performance of hybrids. The ratio of GCA/SCA could be the measure for understanding the inheritance of a particular trait. The RCA effects were significant for plant height, panicle length, number of secondary branches per primary branch and grain yield per plant. This indicates the contribution of the cytoplasmic gene effect and the nuclear gene effect on these traits, as reported by [21]. Therefore, care should be taken in fixing the male and female parents when a hybridization program is taken up to improves these traits, and desirable types should be used as a female parent [53,54]. 

The additive gene action is predominant in most panicle-related traits except panicle length, which shows non-additive gene action. The ratio of GCA/SCA variances explained the majority of gene action responsible for the inheritance of particular traits. The number of spikelets per panicle and number of filled grains per panicle had high additive gene action and a GCA/SCA variance of more than one, which is similar to the previous reports [12,55,56]. For traits showing high additive gene action, the selection method of breeding has been suggested to develop pure lines with desirable characters, i.e., pure line selection and pedigree selection [57,58,59]. In the case of grain yield per plant and the number of productive tillers having high non-additive components, hybrid breeding could be a feasible method for enhancing these traits [60], and hence, postponing the selection to later generations is suggested when recombination breeding is adopted.

CB12132, IET 28749, and BPT 5204 were the good general combiners for most panicle-related traits, especially for the number of spikelets per panicle, number of filled grains per panicle, number of primary branches, and number of secondary branches; these genotypes exhibit a high mean with highly positive significant GCA effect. By comparing the general combining ability effect of panicle-related traits in two high-grain-number genotypes, the long panicle type (CB12132) recorded a maximum GCA higher than the short clustering panicle type (IET 28749). The additive gene effect is predominant due to the general combining ability effect, and these genotypes can be utilized as parents for choosing desirable recombinants and transgressive segregants [61]. 

The crosses, viz., BPT 5204 × CB12132 and IET 28749 × CB12132, exhibited high SCA effect and mean for most panicle-related traits such as panicle length, number of spikelets per panicle, number of filled grains per panicle, and number of primary branches. The high SCA effect occurred due to the effect of non-additive gene actions. To exploit the non-additive genetic variance in recombination breeding, Single-seed-descent (SSD) breeding method or postponement of selection to later segregating generations is suggested. For the number of filled grains per panicle, the high SCA effect of its hybrids occurred when both parents had a high GCA effect, possibly due to additive × additive gene interaction. The cross derived from high × low GCA showed non-significant SCA on its hybrids, possibly due to the epistatic effect. It is not necessary to obtain high SCA observed only from the parents that have high × high and high × low GCA, and it is possible also from low × low GCA combination that appreciable positive SCA occurred in its hybrids, which is due to the complementary gene interaction [62]. 

## 4. Materials and Methods

### 4.1. Plant Genetic Material and Experimental Site

A set of 50 rice accessions comprising 24 genetically stabilized cultures, 14 cultivars, 9 landraces, and 3 mutants originating from different parts of India were used in this study (Table S3). The seeds of the accessions were obtained, and an experiment was conducted at the Department of Rice, Tamil Nadu Agricultural University, Coimbatore, India (Elevation of 426.72 m, between 11° 00 N latitude and 77° 00 E longitude). The field experiment was laid out in randomized block design (RBD) with three replications: row-to-row and plant-to-plant spacing of 20 cm and 20 cm, respectively. Standard agronomic practices and plant protection measures were followed throughout the crop growth period.

### 4.2. Experiments 1 and 2

The aim of experiment 1 was to assess the phenotypic diversity of rice accessions; for this purpose, the seeds of 50 rice genotypes were sown during *Rabi* 2019 and *Summer* 2020 and evaluated for panicle- and other yield-related traits. The aim of experiment 2 was to select the best general and specific combiner and identify the gene action for number of filled grains per panicle and other panicle–yield-related traits. For that purpose, six diverse parents were selected based on the results obtained from experiment 1 and subjected to Griffing’s full diallel mating design method 1. The crossing was attempted among the selected six parents in all possible combinations, including reciprocals, and 30 crosses were generated and evaluated along with parents during *Kharif* 2020. 

### 4.3. Evaluation of Morphological Traits

The morphological observations were recorded according to IRRI’s standard evaluation system (SES) [63]. At anthesis time, the first-formed primary panicles tagged in five plants of each replication of each genotype (i.e., 15 panicles/genotype/season) were collected at the physiological maturity stage. Observations on 14 morphological traits, viz., plant height (PH), number of productive tillers (NPT), flag leaf area (FLA), number of spikelets per panicle (NOSPP), number of filled grains per panicle (NFGPP), number of primary branches per panicle (NOPB), number of secondary branches per panicle (NOSB), number of secondary branches per primary branch (NOSBPB), number of spikelets in primary branches (NOSIPB) and number of spikelets in secondary branches (NOSISB), panicle length (PL), length of primary branch (LOPB), thousand-grain weight (TGW), and grain yield per plant (GYP), were recorded in the already-tagged five plants of each replication in experiments 1 and 2. 

### 4.4. Statistical Analysis

Analysis of variance was calculated for 5% significance level, and the combined analysis of variance of the first experiment across two seasons was computed. Morphological traits of rice germplasm were analysed for mean, the critical difference (CD), genotypic coefficient of variation (GCV), phenotypic coefficient of variation (PCV), heritability, and genetic advance as percentages of the mean (GASM) [64,65,66,67]. Assessment of phenotypic diversity in germplasms was explained by Mahalanobis’ generalized distance (D^2^) analysis and principal component analysis method. For experiment 2, Griffing’s diallel analysis method 1–model 1 was followed, and analysis of variance for RBD and combining ability were calculated at a 5% significance level [68]. Windowstat software was used for the combined analysis of two sets of season data for Mahalanobis’ D^2^ analysis and variability calculations (Indostat services, Hyderabad). The R (4.0.2) packages, FactoMineR, and factoextra were used to perform principal components analysis and obtain a biplot. The diallel analysis was done using the R package “DiallelAnalysisR”.

## 5. Conclusions

The present study explained the variability in diverse rice genotypes for various panicle- and yield-related traits. Results from two multivariate techniques over two seasons indicated that CB12132 and IET 28749 from two different clusters with the genetic distance of 3213.377 were the top-performing genotypes for the number of filled grains per panicle and other panicle characters, i.e., number of spikelets and primary and secondary branches. In the case of grain yield, CO (R) 50 and *Sembil priyan* (PCA group 2) had high grain yield along with high NFGPP. The negative association of the number of productive tillers and thousand-grain weight with NFGPP and other panicle traits was found. Among the studied traits, none of the traits showed complete additive or non-additive gene effects. However, the fraction of additive was high in the number of filled grains per panicle and other panicle traits, and non-additive was high in the number of productive tillers and grain yield per plant. Thus, the hybridization followed by a delay in selection to later generations could be a suitable breeding technique to identify the high-NFGPP genotypes. The maternal effect does not affect the grain number per panicle and other panicle characters except panicle length. CB12132 and IET 28749 are the top two general combiners for the number of filled grains per panicle and other panicle characters with a high mean value. These genotypes can be used to identify genes responsible for the desirable panicle characters using QTL mapping strategies. BPT 5204 × CB 12132 and IET 28749 × CB12132 are the best hybrids for the number of filled grains per panicle and grain yield per plant. The information obtained from this study will be utilized for grain yield improvement in rice breeding programs.

## Figures and Tables

**Table 1:** Pooled estimates of mean and variability of fifty diverse rice genotypes for fourteen panicle- and yield-related traits.

Genotypes	PH	NPT	FLA	PL	NOSPP	NFGPP	NOPB	NOSB	NOSBPB	NOSIPB	NOSISB	LOPB	TGW	GYP
*Sembil priyan*	163.5	12	39.02	28.3	210.5	202	11.5	37.5	3.26	67.5	143	11.25	20.4	45.38
*Karuka*	141	24	39.15	27.95	232	210	14.5	41.5	2.865	90.5	141.5	11.50	15.2	40.42
*Kallukar*	98	11	62.19	25.1	262	239	12.5	55	4.395	66.5	195.5	11.80	23.4	33.025
*Kalarkar*	123	21	33.64	27.3	131	120	7.5	27	3.6	34	97	10.85	21	31.625
*T396 Periya Samba*	113	19.5	26.95	23.8	152	144.5	8.5	23.5	2.765	38.5	113.5	8.50	19.6	17.48
*Nootripathu*	109.5	19	32.34	27.25	124	115	7.5	25	3.335	46.5	77.5	11.05	27.9	13.46
*Muttakar*	77	42.5	35.46	17.05	152.5	145	10	28.5	2.815	42	110.5	6.90	21.4	12.055
*Kothamalli samba*	154	15.5	45.13	28.85	283	257.5	17.5	55	3.14	113	170	11.80	17.3	48.935
*Katta samba*	127.5	16	49.73	28.95	189.5	177.5	14	39	2.785	82.5	107	10.55	21.25	25.75
CO 52	105.5	15	30.45	27.75	285.5	260.5	13.5	60	4.445	67.5	218	8.85	22.5	33.5
IET 29519	89.5	14	31.95	22	116	105	10	26	2.4	52	64	10.75	13.7	25.4
*Mikkuruvai*	134.5	24	33.47	25.15	125.5	119.5	8.5	19.5	2.3	49.5	76	10.60	23.3	35.6
IW Ponni	107.5	18.5	29.25	23.25	185	176.5	12.5	40.5	3.24	58	127	10.50	15	31.305
IARI 3	104.5	18.5	19.22	21.85	156.5	150.5	11.5	31	2.71	23.5	133	10.85	16.9	18.015
IET 28835	93.5	14	24.05	23	102	92.5	9	17	1.89	55.5	46.5	11.00	24	19.075
IET 28834	101	11	17.68	25.55	122.5	112	11.5	27	2.345	57.5	65	9.50	22.4	11.775
ADT (R) 48	74.5	18	12.75	23.75	180	172	14.5	41	2.835	50.5	129.5	8.75	18.4	21.5
BPT 5204	93	14.5	28.88	22.25	207	194.5	11	38	3.455	75	132	9.00	14.05	24.415
CB12132	112.5	16.5	44.61	28.9	430	409.5	15	87	5.97	73	357	13.40	15.9	37
IET 28749	102	15.5	42.04	18.5	417.5	389.5	17	78	4.59	69	348.5	8.50	17.2	31.8
CO 51	77	17	29.36	22	149	141.5	9	32	3.555	50	99	10.50	20	25.6
CO (R) 50	107.5	23.5	42.69	29.25	300	276.5	14	63	4.5	81.5	218.5	13.90	21.3	52.5
ADT 37	83	13	25.06	22.75	217	202.5	9	42.5	4.72	33.5	183.5	11.25	16.5	25.5
MDU 5	75.5	20.5	18.36	27	140	127	10.5	28.5	2.725	37.5	102.5	14.35	21.8	16.5
ADT 45	80.5	17.5	17.78	25.6	128.5	117.5	8	28.5	3.565	45.5	83	11.80	17.9	24.79
ADT 43	80.5	16.5	23.16	24	203	193	8	42.5	5.315	47.5	155.5	12.50	15.1	21.355
TRY 2	95.5	17	19.41	25.5	158.5	149.5	10	35.5	3.55	61	97.5	12.85	30.6	33.635
IET 29537	88	10.5	28.15	25.9	385.5	344	18.5	81.5	5.765	102	283.5	8.70	12	34.83
CRAC 3998-43-1	71	11	31.20	21.95	286	267.5	15	65.5	4.37	83	203	10.55	18.2	15.71
IET 29494	93.5	18.5	30.27	22.65	264.5	239.5	13	60.5	4.655	58	206.5	10.60	16.4	31.745
IET 29530	95.5	15	26.10	25.85	207	197	13	46	3.535	72	135	9.60	24.9	35.18
NWGR-16022	87	10	51.30	22.4	354	326	12	72.5	6.04	51	303	10.65	12.3	22.79
IET 29529	101.5	8.5	36.09	26.75	392.5	345	16	71.5	4.47	67	325.5	12.15	24.9	26.955
IET29523	75	13	30.71	23	292	252	16	65	4.0625	84	208	11.85	15.4	20.33
IET 29504	124.5	11	31.76	29	220	205	12.5	42	3.355	69	151	15.00	15.8	19.2
IET 29539	81	18	29.36	21.15	340	315	17	72	4.24	76.5	263.5	11.85	15.9	24.995
IET 29516	85.5	14.5	32.18	21.75	166	158	10.5	40	3.81	42	124	11.90	23.1	39.33
IET 29495	98	10.5	33.92	22.8	153.5	143.5	10.5	38	3.61	51.5	102	14.20	22.3	37.51
WGL-1283	103	15	36.15	24	240	215	15.5	56	3.61	66	174	9.60	20.12	42.355
BPT 3082	126	18.5	27.32	26	276	248.5	12.5	61.5	4.915	57.5	218.5	11.55	15.9	24.25
KNM 7714	93.5	27	38.53	27.6	380	360	16	76	4.75	106.5	273.5	13.40	13.1	37.64
NP 9253-13	105	17.5	36.94	23.9	225	206	14.5	51	3.515	75	150	9.50	13	30.485
ADT 49	114	21.5	31.06	25.55	268.5	249.5	13.5	67.5	5	56	212.5	11.55	13.2	28.205
KNM 7715	104.5	19	37.74	26.75	333	307.5	13.5	62	4.59	69.5	263.5	10.15	12	41.25
IET 29492	100	19.5	20.72	25	218	207	14	38.5	2.75	86	132	10.00	14.7	30
GNV 1974	95	18	32.91	22.9	263	246	10	47.5	4.75	56.5	206.5	12.00	17	34.51
RNR 29094	100	13.5	30.81	26.5	289	267.5	11.5	52.5	4.565	61.5	227.5	13.50	11.8	17.935
BPT 3059	112.5	10.5	35.46	24.85	278.5	247	14.5	59.5	4.095	71	207.5	11.60	16.3	22.625
Anna (R) 4	95.5	15	32.03	25	197.5	187.5	14.5	46.5	3.205	55	142.5	10.50	25.78	24.15
ACM- 20001	30	35	3.50	8.5	40	30.5	7.5	10.5	1.405	22	18	7.50	16.508	3.5
Maximum	163.5	42.5	62.19	29.25	430	409.5	18.5	87	6.04	113	357	15.5	30.6	52.5
Minimum	30	8.5	3.5	8.5	40	30.5	7.5	10.5	1.405	22	18	6.70	11.8	3.5
Pooled Mean	100.07	17.11	32.13	24.45	228.59	211.29	12.35	47.06	3.76265	62.14	166.45	11.02	18.690	28.06
CD @ 5%	7.02	4.74	1.567	1.60	19.11	21.42	1.70	6.74	0.42	8.96	15.88	1.203	0.920	11.13
CD @ 1%	9.36	6.32	2.089	2.13	25.49	28.56	2.26	8.99	0.55	11.95	21.17	1.605	1.226	14.84
GCV (%)	21.79	34.15	14.19	13.96	34.35	36.61	22.76	37.74	27.07	30.78	45.71	15.36	24.132	33.02
PCV (%)	22.32	36.82	14.54	14.63	39.30	38.99	24.15	39.32	27.62	32.03	47.47	16.29	24.25	38.41
h^2^ (bs) (%)	95.32	86.01	95.54	91.04	76.41	88.18	88.81	92.11	96.05	92.36	92.72	88.50	98.98	74.05
GASM (%)	43.83	65.24	28.53	27.44	61.85	70.83	44.18	74.61	54.64	60.93	90.67	29.83	49.46	58.47

(PH, plant height; NPT, number of productive tillers; FLA, flag leaf area; PL, panicle length; NOSPP, number of spikelets per panicle; NFGPP, number of filled grains per panicle; NOPB, number of primary branches per panicle; NOSB, number of secondary branches per panicle; NOSBPB, number of secondary branches per primary branch; NOSIPB, number of spikelets in primary branches; NOSISB, number of spikelets in secondary branches; LOPB, length of the primary branch; TGW, thousand-grain weight; GYP, grain yield per plant; CD, critical difference; GCV, genotypic coefficient of variation; PCV, phenotypic coefficient of variation; h^2^(bs), broad sense heritability; GASM, genetic advance as percentage of mean.)

**Table 2:** Clustering of 50 rice genotypes for 14 panicle- and yield-related traits computed by Mahalanobis’ D^2^ analysis.

Clusters	Number of Genotypes	Details of Genotypes
Cluster I	29	*Sembil priyan, Karuka, Mikkuruvai*, IET 29504, *Kothamalli samba, T396 Periya Samba*, BPT 3082, NP 9253-13, WGL-1283, IW Ponni, IET 29530, Anna (R) 4, ADT 49, IARI 3, IET29523, *Kallukar*, IET 29492, CO 52, BPT 5204, ADT 37, IET 29516, CO 51, ADT (R) 48, *Muttakar*, IET 29494, GNV 1974, BPT 3059, RNR 29094, CO (R) 50
Cluster II	11	*Kalarkar, Nootripathu, Katta samba*, IET 28835, IET 28834, MDU 5, *ADT* 45, ADT 43, TRY 2, IET 29495, IET 29519
Cluster III	7	CB12132, CRAC 3998-43-1, NWGR-16022, IET 29529, IET 29539, KNM 7714, KNM 7715,
Cluster IV	1	IET28749
Cluster V	1	IET 29537
Cluster VI	1	ACM-20001

**Table 3:** The cluster mean value of six clusters computed from Mahalanobis’ D^2^ analysis using 50 germplasm for 14 panicle- and yield-related traits

Clusters	PH	NPT	FLA	PL	NOSPP	NFGPP	NOPB	NOSB	NOSBPB	NOSIPB	NOSISB	LOPB	TGW	GYP
Cluster I	106.052	19.172	32.190	24.755	222.983	204.052	12.397	46.017	3.707	61.328	161.655	10.869	18.441	29.538
Cluster II	98.046	16.727	27.553	25.586	143.500	133.227	9.636	30.318	3.219	52.273	91.227	11.764	21.946	23.850
Cluster III	93.000	15.714	38.375	25.071	359.357	332.929	14.929	72.714	4.919	75.214	284.143	11.736	16.000	28.831
Cluster IV	102.000	15.500	42.150	18.500	417.500	389.500	17.000	78.000	4.590	69.000	348.500	8.500	17.500	31.800
Cluster V	88.000	10.500	28.180	25.900	385.500	344.000	18.500	106.500	5.765	102.000	283.500	8.700	12.450	34.830
Cluster VI	30.000	35.000	3.475	8.500	40.000	30.500	7.500	10.500	1.405	22.000	18.000	7.500	16.405	4.500

Note: PH, plant height; NPT, number of productive tillers; FLA, flag leaf area; PL, panicle length; NOSPP, number of spikelets per panicle; NFGPP, number of filled grains per panicle; NOPB, number of primary branches per panicle; NOSB, number of secondary branches per panicle; NOSBPB, number of secondary branches per primary branch; NOSIPB, number of spikelets in primary branches; NOSISB, number of spikelets in secondary branches; LOPB, length of primary branch; TGW, thousand-grain weight; GYP, grain yield per plant.

**Table 4:** Average inter (above diagonal) and intra (diagonal) cluster distances of six clusters computed from Mahalanobis’ D^2^ analysis using 50 rice germplasm for 14 panicle- and yield-related traits.

	Cluster I	Cluster II	Cluster III	Cluster IV	Cluster V	Cluster VI
Cluster I	635.2865	1309.547	3888.638	11,524.82	7891.748	3565.379
Cluster II	-	750.9652	7287.658	17,413.5	12,616.88	5179.511
Cluster III	-	-	1244.3348	3213.377	2119.803	3596.254
Cluster IV	-	-	-	0	1569.969	8143.019
Cluster V	-	-	-	-	0	5385.813
Cluster VI	-	-	-	-	-	0

**Table 5:** The eigenvalue, percentage of variation, cumulative percentage, and eigenvector value for the first 10 principal components.

Particulars	PC1	PC2	PC3	PC4	PC5	PC6	PC7	PC8	PC9	PC10
Eigen value	6.627	2.147	1.321	0.968	0.762	0.703	0.542	0.42	0.25	0.14
Variability (%)	47.333	15.339	9.432	6.915	5.445	5.021	3.874	2.999	1.783	1.003
Cumulative (%)	47.333	62.672	72.104	79.02	84.464	89.486	93.36	96.358	98.141	99.144
PH	−0.01	0.923	0.06	−0.027	0.121	0.012	0.204	0.215	0.201	0.015
NPT	−0.135	−0.03	−0.054	0.985	−0.026	−0.067	0.008	−0.008	−0.055	0.005
FLA	0.353	0.266	0.027	−0.01	0.156	0.025	0.177	0.863	0.048	0.023
PL	0.155	0.435	0.331	−0.108	0.21	0.054	0.16	0.063	0.767	0.005
NOSPP	0.928	0.072	0.061	−0.068	0.259	−0.125	0.091	0.149	0.047	0.041
NFGPP	0.926	0.083	0.067	−0.055	0.261	−0.129	0.093	0.139	0.051	0.036
NOPB	0.588	0.066	−0.06	0.025	0.614	−0.046	0.112	0.102	0.019	0.491
NOSB	0.9	−0.036	0.059	−0.05	0.285	−0.114	0.152	0.086	0.06	0.137
NOSBPB	0.858	−0.074	0.192	−0.107	−0.09	−0.14	0.109	0.074	0.134	−0.175
NOSIPB	0.349	0.169	0.067	−0.049	0.856	−0.092	0.229	0.144	0.168	−0.021
NOSISB	0.969	0.038	0.052	−0.065	0.071	−0.119	0.044	0.132	0.01	0.052
LOPB	0.145	0.064	0.973	−0.053	0.026	0.007	0.032	0.019	0.149	−0.011
TGW	−0.305	0.016	0.01	−0.077	−0.076	0.945	0.031	0.019	0.03	−0.008
GYP	0.212	0.225	0.043	0.012	0.203	0.035	0.909	0.154	0.102	0.022

**Table 6:** Analysis of variance for RBD and combining ability effects for diallel analysis method I model 1.

Particulars	Mean Sum of Squares							GCA/SCA
	RBD			Combing Ability				
	Replication	Genotypes	Error	GCA	SCA	RCA	Error	
Degrees of Freedom	2	35	70	5	15	15	35	-
PH	8.337	259.010 **	12.015	755.14 **	36.370 **	14.093 **	6.007	2.06
NPT	0.031	20.660 **	7.524	30.329 *	12.206 *	1.788	3.762	0.26
FLA	127.044	311.9755	22.0256	778.8348 *	93.0763 *	11.2841	11.0128	0.780
PL	2.495	29.355 **	2.935	40.138 *	10.781 *	10.087 *	1.467	0.35
NOSPP	5373.389	27,462.184 **	1983.732	73,557.953 *	6132.930 *	1386.981	991.866	1.181
NFGPP	6815.281	24,995.463 **	2216.160	66,991.797 *	5557.886 *	1272.894	1108.080	1.23
NOPB	10.764	21.775 **	3.045	51.002 *	5.445 *	2.959	1.522	1.05
NOSB	113.351	718.196 **	52.101	2120.779 *	97.240 *	33.729	26.050	2.45
NOSBPB	0.009	1.350 **	0.150	3.886 *	0.131	0.149 *	0.075	5.67
NOSIPB	7.897	477.919 **	60.232	1257.444 *	82.637 *	55.788	30.116	1.95
NOSISB	5350.410	22,803.498 **	1639.771	58,634.324 *	5650.208 *	1409.104	819.88	1
LOPB	0.299	4.654 **	0.628	11.391 *	1.029 *	0.604	0.314	1.29
TGW	0.1569	29.9457	0.3921	84.863 **	6.0763 **	0.5723	0.1960	1.1999
GYP	9.297	67.758 **	6.727	96.108 *	32.275 *	14.740 *	3.363	0.27

Note: PH, plant height; NPT, number of productive tillers; FLA, flag leaf area; PL, panicle length; NOSPP, number of spikelets per panicle; NFGPP, number of filled grains per panicle; NOPB, number of primary branches per panicle; NOSB, number of secondary branches per panicle; NOSBPB, number of secondary branches per primary branch; NOSIPB, number of spikelets in primary branches; NOSISB, number of spikelets in secondary branches; LOPB, length of the primary branch; TGW, thousand-grain weight; GYP, grain yield per plant. * and ** indicate significance at the 5% and 1% levels, respectively).

**Table 7:** General combining ability effects of parental genotypes to 14 panicle- and yield-related traits.

Parents	PH	NPT	FLA	PL	NOSPP	NFGPP	NOPB	NOSB	NOSBPB	NOSIPB	NOSISB	LOPB	TGW	SPY
IET 28834	5 **	−1.271 *	−4.9228 **	−0.817 *	−70.361 **	−66.799 **	−1.084 **	−10.132 **	−0.473 **	−4.741 **	−66.704 **	−0.19 ^ns^	2.719	−1.229 *
IET 28835	1.188 ^ns^	−1.313 *	−4.3907 **	−1.334 **	−64.278 **	−61.299 **	−1.562 **	−9.965 **	−0.4 **	−4.651 **	−58.356 **	−0.348*	2.765	−1.453 **
ADT (R) 48	−15.438 **	1.479 **	−10.9311 **	−1.769 **	−75.611 **	−71.944 **	−2.743 **	−15.25 **	−0.625 **	−16.157 **	−61.35 **	−1.355 **	1.523	−3.768 **
BPT 5204	−0.417 ^ns^	2.438 **	2.7264 **	0.351 ^ns^	43.472 **	39.972 **	1.528 **	7.396 **	0.274 **	8.843 **	33.88 **	−0.194 ^ns^	−2.976	1.266 *
CB12132	6.063 **	−0.292 ^ns^	8.1956 **	3.31 **	88.514 **	87.41 **	1.528 **	15.354 **	0.718 **	7.634 **	80.255 **	1.562 **	−2.726	0.696 ^ns^
IET 28749	3.604 **	−1.042 *	9.3226 **	0.26 ^ns^	78.264 **	72.66 **	2.333 **	12.597 **	0.504 **	9.072 **	72.275 **	0.525 **	−2.726	4.488 **
SE (gi)	0.6459	0.5111	0.8745	0.1690	8.2994	8.7721	0.3251	1.3450	0.0721	1.4462	7.5456	0.1476	−1.306	0.4833

Note: PH, plant height, NPT, number of productive tillers; FLA, flag leaf area; PL, panicle length; NOSPP, number of spikelets per panicle; NFGPP, number of filled grains per panicle; NOPB, number of primary branches per panicle; NOSB, number of secondary branches per panicle; NOSBPB, number of secondary branches per primary branch; NOSIPB, number of spikelets in primary branches; NOSISB, number of spikelets in secondary branches; LOPB, length of primary branch; TGW, thousand-grain weight; GYP, grain yield per plant. * 5% significance; ** 1% significance; ^ns^ non-significance; SE (gi), standard error for GCA effects of parents.)

## Data Availability

Data are contained within this article and the Supplementary Information.

## Supplementary Materials

The following supporting information can be downloaded at: https://www.mdpi.com/article/10.3390/plants12020250/s1, Table S1: Analysis of variance for fourteen panicle and yield related traits estimated over two seasons, Table S2: Specific combining ability effects of direct and reciprocal hybrids to panicle and yield related traits, Table S3: List of genotypes used for phenotypic diversity for panicle and yield related traits.
