# Supplementary material for: Identification of Newer Stable Genetic Sources for High Grain Number per Panicle and Understanding the Gene Action for Important Panicle Traits in Rice
Source: Plants (Basel). 2023 Jan 5;12(2):250. doi: 10.3390/plants12020250 (PMC9860970; doi:10.3390/plants12020250)
Supplement: Supplementary file 1 [file plants-12-00250-s001.zip › plants-1897164-supplementary.pdf]

Table S1 Analysis of variance for fourteen panicle and yield related traits estimated over two seasons

Characters	Genotypes	Season	Genotypes × Season	Error
Degrees of freedom (DF)	49	1	49	294
Plant height (PH)	259.010 **	645.85**	110.21**	25.65
Number of productive tillers (NPT)	20.660 **	565.70**	15.75**	6.80
Flag leaf area (FLA)	35.60**	245.50**	8.50**	4.52
Panicle length (PL)	29.355 **	266. 26**	6.99**	3.52
Number of spikelets per panicle (NOSPP)	27462.184 **	12443.14**	2268.52**	850.20
Number of grains per panicle (NOGPP)	24995.463 **	15184.17**	2035.40**	675.42
Number of primary branches per panicle (NOPB)	21.775 **	6.61**	2.14*	1.05
Number of secondary branches per panicle (NOSB)	718.196 **	2370.5**	134.52**	45.60
Number of secondary branches per primary branches (NOSBPB)	1.350 **	4.56**	1.56	0.50
Number of spikelets in primary branches (NOSIPB)	477.919 **	354.50**	78.50**	32.50
Number of spikelets in secondary branches (NOSISB)	22803.498 **	8456.50**	1120.60**	265.68
Length of primary branches (LOPB)	3.65**	78.03**	1.56*	1.02
Thousand grain weight (TGW)				
Grain yield per plant (GYP)	67.758 **	2701.09**	65.50**	15.65

Table S2 Specific combining ability effects of direct and reciprocal hybrids to panicle and yield related traits.

Cross Name	ID	D/R	PH	NPT	FLA	PL	NOSPP	NFGPP	NOPB	NOSB	NOSBPB	NOSIPB	NOSISB	LOPB	TGW	GYP
IET 28834 ×	C1	D	-2.792^{ns}	-2.896*	2.922 ^{ns}	-5.431**	54.236**	50.924*	0.611^{ns}	8.222**	0.448**	3.213^{ns}	50.648**	0.346^{ns}	-0.907**	-1.002^{ns}
IET 28835	C2	R	7.5**	0.25^{ns}	-1.94 ^{ns}	8.425**	15.125^{ns}	14.875^{ns}	1.125^{ns}	1.75^{ns}	-0.053^{ns}	9.375*	5.75^{ns}	0.15^{ns}	-0.875**	0.023^{ns}
IET 28834 ×	C3	D	2.458^{ns}	2.438*	4.437 *	0.917^{ns}	25.32^{ns}	22.319^{ns}	0.042^{ns}	4.882^{ns}	0.151^{ns}	3.095^{ns}	16.766^{ns}	-0.622^{ns}	-0.740**	-2.247*
ADT (R) 48	C4	R	0.875^{ns}	2.125^{ns}	-0.115^{ns}	-0.688^{ns}	-3.125^{ns}	-3.625^{ns}	-0.125^{ns}	0.875^{ns}	0.12^{ns}	-3.75^{ns}	8.375^{ns}	0.375^{ns}	0.250^{ns}	1.468^{ns}
IET 28834 ×	C5	D	1.938^{ns}	1.854^{ns}	-3.342^{ns}	0.884^{ns}	5.236^{ns}	7.403^{ns}	2.896**	0.611^{ns}	-0.199^{ns}	10.72**	-3.838^{ns}	0.379^{ns}	1.085**	3.204**
BPT 5204	C6	R	2.375^{ns}	-0.5^{ns}	1.282^{ns}	0.1^{ns}	17.375^{ns}	19.375^{ns}	3.25**	1.25^{ns}	-0.275^{ns}	7.375^{ns}	10^{ns}	0.238^{ns}	0.075^{ns}	-2.603^{ns}
IET 28834 ×	C7	D	-3.667*	0.833^{ns}	-8.653**	-0.412^{ns}	-88.056**	-85.785**	-1.729*	-11.097**	-0.297^{ns}	-9.197**	-77.338**	-1.077**	1.135**	0.704^{ns}
CB12132	C8	R	0.25^{ns}	1.5^{ns}	-1.620^{ns}	-0.313^{ns}	3.125^{ns}	3.375^{ns}	0.625^{ns}	-2.75^{ns}	-0.38^{ns}	6.5^{ns}	-3.375^{ns}	0.588^{ns}	0.475^{ns}	6.703**
IET 28834 ×	C9	D	0.417^{ns}	0.208^{ns}	-3.598^{ns}	1.263^{ns}	-9.931^{ns}	-7.41^{ns}	0.173^{ns}	-1.508^{ns}	-0.041^{ns}	2.491^{ns}	-11.734^{ns}	0.386^{ns}	1.914**	2.16^{ns}
IET 28749	C10	R	-0.375^{ns}	-0.375^{ns}	-1.347^{ns}	-0.688^{ns}	5.5^{ns}	5^{ns}	1.918*	1.668^{ns}	-0.223^{ns}	3.625^{ns}	4.75^{ns}	0.463^{ns}	0.375^{ns}	2.18^{ns}
IET 28835 ×	C11	D	1.521^{ns}	0.354^{ns}	2.995^{ns}	0.922^{ns}	6.861^{ns}	4.444^{ns}	-0.354^{ns}	3.09^{ns}	0.053^{ns}	-2.62^{ns}	7.919^{ns}	-0.579^{ns}	0.339^{ns}	-1.205^{ns}
ADT (R) 48	C12	R	1.375^{ns}	-0.25^{ns}	1.255^{ns}	0.475^{ns}	0.25^{ns}	1^{ns}	0^{ns}	-1^{ns}	-0.105^{ns}	-1.375^{ns}	-0.375^{ns}	-0.485^{ns}	-0.325^{ns}	0.705^{ns}
IET 28835 ×	C13	D	1.125^{ns}	0.646^{ns}	-0.839^{ns}	-0.212^{ns}	-14.347^{ns}	-11.097^{ns}	-0.125^{ns}	-3.056^{ns}	-0.097^{ns}	2.38^{ns}	-20.81^{ns}	0.575^{ns}	1.364**	1.543^{ns}
BPT 5204	C14	R	2.75^{ns}	-1^{ns}	-3.137^{ns}	-0.913^{ns}	-2.125^{ns}	-4.125^{ns}	0.75^{ns}	-4^{ns}	-0.435*	6.375^{ns}	-11.875^{ns}	1.05*	-0.150^{ns}	-3.713**
IET 28835 ×	C15	D	-3.854*	-0.875^{ns}	-5.716**	-0.333^{ns}	-61.889**	-61.785**	-0.125^{ns}	-11.264**	-0.58**	-2.287^{ns}	-63.06**	0.382^{ns}	0.989**	-0.792^{ns}
CB12132	C16	R	-2^{ns}	-0.75^{ns}	-1.165^{ns}	-0.75^{ns}	-15.875^{ns}	-14.625^{ns}	-0.5^{ns}	-3^{ns}	-0.15^{ns}	-3^{ns}	-15.5^{ns}	0.638^{ns}	0.925**	3.948**
IET 28835 ×	C17	D	3.229*	-0.875^{ns}	-3.713^{ns}	2.867**	-8.389^{ns}	-4.535^{ns}	0.694^{ns}	0.618^{ns}	0.009^{ns}	6.651*	-2.831^{ns}	-0.444^{ns}	1.343**	-3.335**
IET 28749	C18	R	-2.125^{ns}	-1.5^{ns}	0.130^{ns}	0.275^{ns}	-6.875^{ns}	-5.125^{ns}	-0.375^{ns}	1.375^{ns}	0.175^{ns}	-2.375^{ns}	12.25^{ns}	0.325^{ns}	0.050^{ns}	-2.125^{ns}
ADT (R) 48 ×	C19	D	-3.75*	3.979**	-4.071*	-0.226^{ns}	-64.389**	-59.951**	-2.069**	-6.521*	-0.047^{ns}	-9.239**	-52.692**	-0.131^{ns}	-0.194^{ns}	-0.35^{ns}
BPT 5204	C20	R	-1.25^{ns}	1.375^{ns}	0.840^{ns}	0.663^{ns}	-8.75^{ns}	-8.875^{ns}	0.125^{ns}	-0.75^{ns}	-0.115^{ns}	-2.75^{ns}	-6^{ns}	-0.113^{ns}	0.050^{ns}	1.695^{ns}
ADT (R) 48 ×	C21	D	-1.854^{ns}	-0.292^{ns}	-6.483**	-1.06^{ns}	-7.431^{ns}	-8.014^{ns}	-0.694^{ns}	-1.104^{ns}	0.22^{ns}	5.345^{ns}	-10.442^{ns}	0.901*	0.031^{ns}	3.108**
CB 12132	C22	R	-3.875*	-0.375^{ns}	0.922^{ns}	0.463^{ns}	-75.75**	-76**	-1.25^{ns}	-13.125**	-0.72**	-4.875^{ns}	-70.875**	-0.85*	1.175**	-2.738*
ADT (R) 48 ×	C23	D	-1.646^{ns}	-4.292**	-4.035*	2.015**	-70.306**	-63.639**	-1.75*	-6.972*	-0.004^{ns}	-2.093^{ns}	-69.588**	0.013^{ns}	2.960**	-6.236**
IET 28749	C24	R	0.625^{ns}	-0.125^{ns}	-1.192^{ns}	0.138^{ns}	-19.875^{ns}	-21.875^{ns}	-0.5^{ns}	-1.75^{ns}	-0.033^{ns}	0.125^{ns}	-20^{ns}	-0.85*	-0.175^{ns}	0.48^{ns}
BPT 5204 ×	C25	D	2.375^{ns}	-2.375*	9.276**	2.582**	100.361**	100.57**	1.285^{ns}	11.875**	0.238^{ns}	-0.655^{ns}	102.204**	0.715*	-0.394^{ns}	-8.794**
CB 12132	C26	R	0.625^{ns}	0.5^{ns}	-2.010^{ns}	1.025^{ns}	-14.625^{ns}	-17.5^{ns}	-1^{ns}	-0.5^{ns}	-0.053^{ns}	-1.125^{ns}	-13.5^{ns}	0.125^{ns}	0.000^{ns}	2.6^{ns}
BPT 5204 ×	C27	D	5.583**	0.5^{ns}	4.134*	-0.593^{ns}	31.111^{ns}	11.069^{ns}	0.729^{ns}	5.257^{ns}	0.094^{ns}	2.282^{ns}	26.308^{ns}	0.115^{ns}	-2.665**	1.95^{ns}
IET 28749	C28	R	-1.875^{ns}	-0.125^{ns}	0.550^{ns}	-0.375^{ns}	-25.625^{ns}	4.75^{ns}	-1.5^{ns}	-5.625^{ns}	-0.03^{ns}	-2^{ns}	-23.625^{ns}	-0.388^{ns}	0.450^{ns}	0.445^{ns}
CB12132 ×	C29	D	5.729**	1.104^{ns}	14.833**	-0.301^{ns}	13.319^{ns}	15.382^{ns}	-0.021^{ns}	2.924^{ns}	0.143^{ns}	3.366^{ns}	7.308^{ns}	0.596^{ns}	-2.265**	-0.103^{ns}
IET 28749	C30	R	-2^{ns}	0^{ns}	-7.39**	-0.25^{ns}	-49.375*	-44.5^{ns}	0.5^{ns}	-1.25^{ns}	-0.193^{ns}	10.625**	-60**	0.525^{ns}	0.700*	-0.123^{ns}
SE (Sij)				1.1656	1.9942	0.3853	18.9255	20.0035	0.7414	3.0671	0.1645	3.2977	17.2067	0.3367	0.266	1.1021
SE (Rij)				1.3715	2.3466	0.4534	22.2696	23.5381	0.8724	3.6091	0.1936	3.8804	20.2470	0.3962	0.313	1.2968

TABLE S3 List of genotypes used for morphological diversity for panicle and yield related traits

Sl. No.	Genotype name	Parentage	Type
1	*Sembil priyan*	-	Landrace
2	*Karuka*	-	Landrace
3	*Kallukar*	-	Landrace
4	*Kalarkar*	-	Landrace
5	*T 396 periya samba*	-	Landrace
6	*Nootripathu*	-	Landrace
7	*Muttakar*	-	Landrace
8	*Kothamalli samba*	-	Landrace
9	*Katta samba*	-	Landrace
10	CO 52	CO (R) 50/ BPT 5204	Landrace
11	IET29519	CN 1920 / Savitri // IR 71700-247- 1-1-2	Culture
12	*Mikkuruvai*	-	Culture
13	IW PONNI	-	Variety
14	IARI 3	-	Variety
15	IET 28835	Anjali*3/IR 81896-B-195	Culture
16	IET 28834	IR84984-83-15-481-B/3* Anjali // IR81896-B-B-195/3* Anjali	Culture
17	ADT (R) 48	IET 11412/IR 64	Variety
18	BPT 5204	GEB-24 ×T(N) 1× Mahsuri	Landrace

19	CB12132	CO (R) 50 / CB501	Culture
20	IET 28749	BPT5 204 mutant	Variety
21	CO 51	ADT 43/RR 272-1745	Variety
22	CO (R) 50	CO 43/ ADT38	Variety
23	ADT 37	BG 280-12/PTB 33	Variety
24	MDU 5	*O. glaberrima*/ Pokkali	Variety
25	ADT 45	IR 50/ADT 37	Variety
26	ADT 43	IR 50/ White ponni	Variety
27	TRY 2	RP 825 – 45 – 1- 3 / IR 36	Variety
28	IET 29537	Samba Mashuri*2 / ST 12	Culture
29	CRAC 3998-43-1	Doubled haploid of 27P63	Culture
30	IET 29494	ADT 49 / MTU 1150	Culture
31	IET 29530	Doubled haploid of 27P63	Culture
32	IET 29499	GR-11 / IET 19791(26-3-1-1-1-1-1)	
33	IET 29529	RP 5977 / BPT 5204	Culture
34	IET 29523	R 1033-2559-1-1 / Gopal bhog	
35	IET 29504	BPT 5204 Mutant	Culture
36	IET 29539	MTU 1156 / MTU 1081	Variety
37	IET 29516	ADT 43 / BPT 5204	Culture
38	IET 29495	MTU 1010 / RPHR 1005	Culture
39	WGL-1283	WGL-14 / JGL -17025	Culture
40	BPT 3082	BPT 5204 / MTU 1075	Culture

41	KNM 7714	RNR 15048 / GNV -8-89 // WGL 32183	Culture
42	NP 9253-13	NP 14 / NP 4201	Culture
43	ADT 49	CR 1009/J.Samba	Variety
44	KNM 7715	RNR 15048 / GNV - 8 – 89 // WGL 32183	Culture
45	IET 29492	CSR 27 / BPT 5204	Culture
46	GNV 1974	IET 24716 / NLR 34449	Culture
47	RNR 29094	BPT-5204 / BM-7 (BBM-1) // BPT5204 / BM-71(BBM-2)	Culture
48	BPT 3059	MTU 1061 / IR 78585-64-2-4-3	Culture
49	ANNA (R) 4	Pantdhan 10 X IET 9911	Variety
50	ACM 20001	Anna (R) 4 mutant	Mutant
